# Alterations in Corneal Nerve Structure and Function in Prediabetes

**DOI:** 10.1155/jdr/4586856

**Published:** 2025-04-12

**Authors:** Jun Ma, Junfang Zhang, Mengjia Tan, Min Ji, Jianfeng Yu, Huaijin Guan

**Affiliations:** ^1^Department of Ophthalmology, Medical School of Nantong University, The Affiliated Hospital of Nantong University, Nantong, Jiangsu, China; ^2^Department of Ophthalmology, Yancheng First People's Hospital, Affiliated Hospital of Medical School, Nanjing University, Yancheng, Jiangsu, China

**Keywords:** corneal nerve, corneal neuropathy, diabetes, in vivo confocal microscopy, prediabetes

## Abstract

**Background:** Although prediabetes increases the risk of developing diabetes, its role in neuropathy remains unclear. We aim to assess alterations in the corneal nerve structure and function in prediabetes and risk factors for corneal nerve loss.

**Methods:** An examination of IVCM and corneal sensitivity was conducted on a cohort of 75 participants, comprising 23 controls, 32 prediabetes, and 20 Type 2 diabetes. Semiautomatic analysis was employed to quantify the corneal nerve fiber length (CNFL), corneal nerve fiber density (CNFD), and dendritic cell (DC) density.

**Results:** CNFL and CNFD were lower in prediabetes and Type 2 diabetes than in the controls, and they were associated with DC density. CNFL and CNFD were lower in Type 2 diabetes than in prediabetes. DC density was higher in prediabetes and Type 2 diabetes than in controls. However, there were no differences in corneal sensitivity between controls and prediabetes. Multivariable regression analysis demonstrated an association between reduced CNFL and age, BMI, fasting plasma glucose (FPG), and uric acid (UA) levels in prediabetes. In Type 2 diabetes, age, HbA1c, blood urea nitrogen (BUN), creatinine (Cr), and triglyceride levels exhibited associations with reduced CNFL.

**Conclusions:** Corneal nerve damage was detected in prediabetes using IVCM. The patients with prediabetes showed signs of nerve structure damage, and the corneal nerve structure damage occurred before the nerve function changes. Immune cells also participate in the occurrence and development of DCN and are related to the corneal neuropathy. Understanding the corneal nerve fiber condition through IVCM may prove crucial in monitoring prediabetic neuropathy.

## 1. Introduction

Diabetes mellitus (DM) is a major global public health concern [1], with diabetic peripheral neuropathy (DPN) being the most common long-term complication, affecting at least 50% of patients [2]. DPN typically presents with sensory loss and pain in small unmyelinated C fibers [3], and the corneal manifestation is referred to as diabetic corneal neuropathy (DCN). Prediabetes is defined as a state where blood sugar levels do not meet the criteria for diabetes but show abnormal metabolism of carbohydrates. This state is characterized by impaired fasting glucose (IFG) (or FPG levels ranging from 5.6 to 6.9 mmol/L), impaired glucose tolerance (IGT) (or 2-h postprandial glucose (PPG) levels between 7.8 and 11.0 mmol/L), and/or HbA1c of 5.7%–6.4% [4]. Although prediabetes is recognized as a risk factor for diabetes and heart disease, its contribution to neuropathy development remains uncertain.

**Figure 1:**
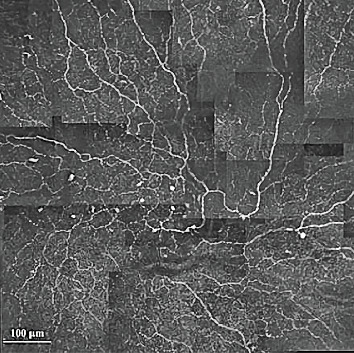
IVCM images from the vortex structure region demonstrating the highly complex pattern of nerves.

In vivo confocal microscopy (IVCM) serves as a rapid, nonintrusive imaging technique that facilitates the objective evaluation of early degeneration [5], allowing subsequent regeneration of the corneal nerves after reducing risk factors [6]. IVCM allows visualization of all corneal layers, including the epithelium, subbasal layer, Bowman's layer, stroma, and endothelium. Of these, the subbasal layer is particularly crucial as it offers clear visualization of epithelial immune dendritic cells (DCs) and the subbasal nerve plexus (SNP) [7–10]. The vortex structure of SNP, slightly nasal to the corneal apex, serves as a distinctive anatomical landmark for consistent corneal scanning (Figure 1). Davidson et al. [11] demonstrated that nerve loss in the vortex structure region precedes nerve loss in the central cornea. Corneal DCs act as primary antigen-presenting cells, playing a pivotal role in activating the immune system on the ocular surface while regulating inflammation [12–14]. Due to the diversity seen in epithelial DCs and SNP across various corneal and ocular surface disorders, including dry eye disease, neurotrophic keratopathy, infectious keratitis, diabetes, and contact lens use [5, 7, 15–18], it is crucial to utilize IVCM for the measurement of these features. However, a potential limitation of IVCM is that only a relatively small area of the corneal tissue is scanned. To overcome this limitation, our study employed corneal SNP mosaics synthesized from a standard single image to enhance IVCM technology.

Investigating neuropathy in prediabetes is pivotal, as it can provide insights into the early stages of diabetic neuropathy and underscore that neuropathy may manifest even in the presence of minimal metabolic derangement. Our objective was to evaluate changes in the corneal nerve structure and function in prediabetes and investigate the diagnostic potential of IVCM for corneal neuropathy, along with exploring risk factors associated with corneal nerve loss.

## 2. Subjects and Methods

### 2.1. Study Subjects

This study involved the analysis of 75 patients with cataracts who received treatment at the Affiliated Hospital of Nantong University between September 2022 and August 2023. The participants consisted of 23 controls (40 eyes), 32 prediabetic patients (60 eyes), and 20 Type 2 diabetic patients (40 eyes); ten eyes met the exclusion criteria and were excluded. Prediabetes was defined by the presence of IFG, IGT, and/or HbA1c ranging from 5.7% to 6.4% [4]. Exclusion criteria encompassed individuals with a history of contact lens use, corneal disease, glaucoma, uveitis, fundus diseases unrelated to diabetes, malignancy, other causes of neuropathy, current or active diabetic foot ulceration, B12 or folate deficiency, chronic renal or liver failure, or severe systemic diseases. This study adhered to the principles outlined in the Declaration of Helsinki and received approval from the Ethics Committee of the Affiliated Hospital of Nantong University. Prior to participation, informed consent was obtained from each participant.

### 2.2. Physical Examination and Laboratory Measurements

For every patient, a comprehensive medical assessment was conducted, covering parameters such as body height, body weight, medical history, and blood sampling. All participants underwent assessments for BMI, HbA1c, FPG, lipid fractions (total cholesterol and triglycerides), BUN, Cr, and UA.

### 2.3. IVCM

All participants underwent IVCM using the Heidelberg Retinal Tomograph III Rostock Cornea Module (Heidelberg Engineering GmbH, Heidelberg, Germany). To anesthetize each eye, a drop of proparacaine hydrochloride (Alcaine; S.A. Alcon-Couvreur N.V., Puurs, Belgium) was administered. Subsequently, a large drop of lubricant (2 mg/g ganciclovir ophthalmic gel; Likeming, China Meheco Keyi Pharma Co., Ltd., Hubei, China) was applied to the lens tip before attaching the disposable cap (TomoCap). Patients were instructed to focus on a target, and the machine was systematically moved to enable the examiner to visualize the vortex structure of SNP. In the “section” mode, the images were captured within a 2-to-3-mm radius of the vortex structure. Approximately 100–200 clear images were collected for each examined eye. Overlapping portions of the images were stacked using Photoshop 2022 image processing software (Adobe Inc., United States), and centered images on the vortex structure were formed after clipping 800 × 800 *μ*m-sized images. For analysis, the ImageJ image analysis system (National Institute of Mental Health, United States) was employed to assess the CNFL (total length of the main nerves and nerve branches per square millimeter (/mm^2^)) and CNFD (number of main nerves and nerve branches per square millimeter (mm/mm^2^)). Using the ImageJ software, DCs in the vortex area were counted, and DC density (no./ mm^2^) was calculated based on the image size. Immature cells were defined as DCs < 50 *μ*m in length without dendritic structures, while mature cells were those with a length > 50 *μ*m and exhibiting dendritic structures.

### 2.4. Measurement of Corneal Sensitivity

Corneal sensitivity was assessed using a Cochet-Bonnet esthesiometer (Luneau Ophthalmologie Chartres, Cedex, France). This device features a flexible nylon monofilament measuring 6.0 cm in length that can be adjusted accordingly. The filament, when fully extended, maintains a soft texture, whereas when retracted into the headpiece, it stiffens, creating a pressure gradient ranging from 11 to 200 mg/mm^2^. Measurements were conducted on the nasal side of the cornea, precisely 2 mm from the central cornea. Initiating with the longest filament length of 6.0 cm, which corresponded to the lowest possible pressure, the filament length was gradually reduced in 2-mm increments until the patient could discern the sensation. The point at which the patient recognized sensitivity was recorded, and the results were expressed in terms of filament lengths, measured in millimeters. Each measurement at a specific filament length was repeated three times to minimize potential errors. Higher numerical values from the measurements indicated greater corneal sensitivity.

### 2.5. Statistical Analysis

Statistical analysis was conducted using IBM SPSS v27 (Chicago, IL, United States). Mean ± SD was used for data expression, and normality was assessed. To compare means among groups, one-way ANOVA was conducted for parametric variables, and Kruskal–Wallis test was employed for nonparametric variables. Chi-square test is performed for the categorical variables. Correlation among IVCM data was evaluated using the Pearson correlation coefficient (Spearman's rank correlation coefficient for nonparametric data). The association between IVCM measures and clinical findings was explored using generalized linear models.

## 3. Results

### 3.1. Baseline Characteristics

Significant differences were observed in HbA1c (*p* < 0.001) and FPG (*p* < 0.001), but not in age (*p* = 0.090), sex (*p* = 0.105), BMI (*p* = 0.700), BUN (*p* = 0.483), Cr (*p* = 0.399), UA (*p* = 0.648), total cholesterol (*p* = 0.320), or triglycerides (*p* = 0.279), among the groups (Table 1).

### 3.2. Corneal Nerve Structure and Function

**Figure 2:**
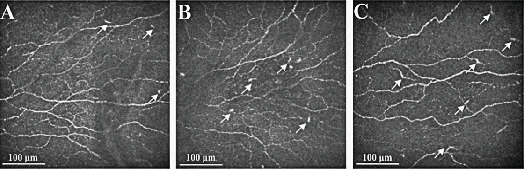
The IVCM from three group subject's images shown that Type 2 diabetes' DCs maturity was higher. (A) Controls; (B) prediabetes; and (C) Type 2 diabetes.

Corneal SNP mosaics of IVCM displayed a significant reduction in CNFL and CNFD in prediabetic and Type 2 diabetic patients. CNFL (all *p* < 0.0001) and CNFD (*p* = 0.0420 and *p* < 0.0001, respectively) were significantly lower in prediabetic and Type 2 diabetic patients than in controls. Furthermore, CNFL (*p* = 0.0370) and CNFD (*p* = 0.0472) were significantly lower in Type 2 diabetic patients than in those with prediabetes. Conversely, DC density was significantly higher in prediabetic and Type 2 diabetic patients than in controls (*p* = 0.0004 and *p* < 0.0001, respectively). Interestingly, no differences were identified in corneal sensitivity between the controls and prediabetes (Table 2). Although no difference in DC density was observed between prediabetic and Type 2 diabetic patients, IVCM images indicated higher DC maturity in Type 2 diabetic patients (Figure 2).

### 3.3. Correlations and Multivariable Regression Analysis

**Figure 3:**
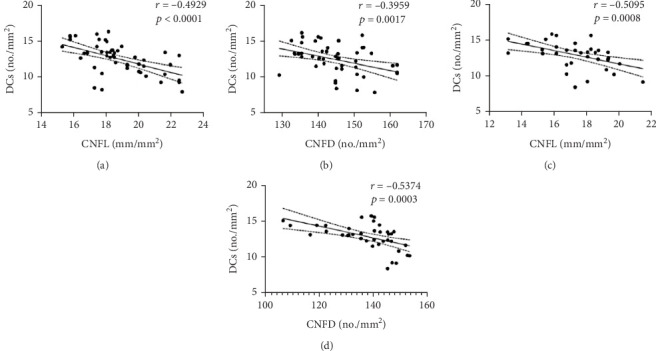
The correlation between CNFL, CNFD, and DCs in prediabetes and Type 2 diabetes. (a) CNFL in prediabetes; (b) CNFD in prediabetes; (c) CNFL in Type 2 diabetes; and (d) CNFD in Type 2 diabetes.

In prediabetic patients, CNFL (*r* = −0.4929, *p* < 0.0001) and CNFD (*r* = −0.3959, *p* = 0.0017) showed a significant negative correlation with DC density. Similar findings were reported in Type 2 diabetic patients (CNFL: *r* = −0.5095, *p* = 0.0008; CNFD: *r* = −0.5374, *p* = 0.0003) (Figure 3).

Multivariable regression analysis unveiled a significant association between reduced CNFL and various factors in prediabetic patients: age (*β* = −0.356, *p* = 0.001), BMI (*β* = 0.531, *p* < 0.001), FPG (*β* = 0.373, *p* = 0.001), and UA levels (*β* = −0.303, *p* = 0.027) were identified as contributing factors. Similarly, in Type 2 diabetic patients, reduced CNFL was significantly associated with age (*β* = −0.472, *p* = 0.010), HbA1c (*β* = −0.922, *p* < 0.001), BUN (*β* = −0.585, *p* < 0.001), Cr (*β* = −0.474, *p* = 0.031), and triglyceride levels (*β* = 0.422, *p* = 0.024) (Table 3).

## 4. Discussion

Throughout the clinical course of DM, DCN affects a substantial percentage of patients, ranging from 47% to 64%. Notably, DPN diagnosis relies on identifying abnormal symptoms and signs, which, unfortunately, are not entirely reliable for detecting early damage to small nerve fibers. The gold standard for assessing small fiber damage is intraepidermal nerve fiber density quantification, which is an invasive procedure [19, 20]. This also led to DCN being often overlooked. DCN is characterized by progressive damage to the corneal nerves, which results in decreased corneal sensitivity. Consequently, DCN patients are more vulnerable to various anterior segment pathological conditions, including dry eye disease and neurotrophic ulcers, which pose a risk for sight-threatening corneal infections [21]. In this context, IVCM is a rapid, nonintrusive imaging technique in ophthalmology for DCN diagnosis.

The association between corneal neuropathy and prediabetes remains a subject of controversy, despite prediabetes being a critical risk factor for the progression to diabetes. Fujimoto et al. [22] demonstrated that nerve conduction study findings were comparable between IGT patients and healthy controls. However, IGT patients reported a higher prevalence of retinopathy and nephropathy than controls. Asghar et al. [23] highlighted significant alterations in the sudomotor function in IGT patients; however, other aspects of cardiac autonomic function and electrophysiology were within normal ranges. Additionally, IGT patients experienced a threefold increase in neuropathic pain. The results of IVCM demonstrated a significant abnormality in corneal nerve structure and 40.5% of subjects with IGT have significant small-fiber damage based on CNFD decrease. Azmi et al. [24] validated that IVCM and skin biopsy show a dynamic worsening or improvement in corneal and intraepidermal nerve morphology in relation to change in glucose tolerance status. Mokhtar et al. [25] indicated that glycaemia-associated corneal neurodegeneration is a continuous process that starts before the onset of Type 2 diabetes. In our study, prediabetic patients exhibited a mean CNFL of 18.64 ± 1.85 mm/mm^2^, consistent with previous findings [23, 24, 26]. IVCM revealed a noticeable reduction in both CNFL and CNFD in prediabetic patients compared with controls, indicating reasonable diagnostic utility for corneal neuropathy. Importantly, IVCM may identify corneal neuropathy at an early stage before the onset of corneal sensory dysfunction, even if corneal sensitivity does not align with early morphological changes in the corneal subbasal nerves.

The interplay between DCs and nerves in neuropathy pathogenesis has been suggested [10]. Nerves influence immune cell activity by releasing cytokines and neuropeptides [27], and resident immune cells express the relevant neuropeptide receptors [28]. DCs express neurotrophic factors that promote corneal reinnervation and corneal denervation [29]. However, the precise role of corneal immune cells in the early stages of diabetes-associated corneal neuropathy remains unclear, with contrasting findings reported between mouse and human studies. Animal and human studies have suggested that DCN [30] is associated with higher DC density in the corneal epithelium. Moreover, diabetes has been reported to reduce corneal DC density in mice, which underpins delayed corneal epithelial wound-healing responses after injury [31]. In our study, DC density was significantly higher in prediabetic and Type 2 diabetic patients than in controls. IVCM images indicated higher DC maturity in Type 2 diabetic patients than in prediabetic patients, whereas no difference was noted in DC density. DC density in prediabetic and Type 2 diabetic patients was correlated with reduced CNFL and CNFD, suggesting a potential role of corneal immune cells in diabetic neuropathy, dependent on the disease type, stage, and duration.

This study highlighted differences in risk factors associated with corneal nerve loss between prediabetic and Type 2 diabetic patients. In prediabetes, corneal nerve loss was associated with age, BMI, FPG, and UA levels, whereas in Type 2 diabetes, it was associated with age, HbA1c, BUN, Cr, and triglyceride levels. These variations in risk factors may stem from differing metabolic states in prediabetes and Type 2 diabetes. HbA1c has been demonstrated to be associated with corneal nerve loss in Type 2 diabetic patients [32]. Furthermore, several interventional studies on Type 2 diabetic patients have demonstrated that a reduction in HbA1c is associated with an improvement in corneal nerve morphology [33–35]. Additionally, the association between older age and reduced CNFL and CNFD reported in this study aligns with the findings reported by Andersen et al. [32].

This study presents some limitations that warrant acknowledgment. First, the study's cross-sectional nature hinders the ability to provide insights into the prospective performance of SNP in prediabetes. Azmi et al. [24] demonstrated that the IGT subjects showed no change in IVCM over 3 years but those who returned to normal glucose tolerance showed an increase in CNFD and CNFL. To elucidate the long-term implications and predictive value of SNP, further longitudinal studies are imperative. Second, the relatively prolonged scanning time required for each cornea, ranging from 20 to 30 min, poses a potential limitation, as the extended duration may cause discomfort to the participants and may not be conducive to the efficient screening of prediabetes. Therefore, alternative methods that are quicker and less burdensome need to be identified to ensure that both patient comfort and system efficiency are prioritized.

In conclusion, this study reveals a significant decrease in corneal nerve fiber parameters in prediabetic patients compared with those with normal blood glucose levels, detected using IVCM. Immune cells also participate in the occurrence and development of DCN and are related to the corneal neuropathy. The information provided by IVCM on the corneal nerve fiber condition is deemed crucial for monitoring DPN.

## Figures and Tables

**Table 1:** Clinical, demographic, and laboratory results in controls, prediabetic patients, and Type 2 diabetic patients (mean ± SD).

	**Controls (** **n** = 23**)**	**Prediabetes (** **n** = 32**)**	**Type 2 diabetes (** **n** = 20**)**	**p** ** value**
Age (years)	64.04 ± 8.37	63.69 ± 7.39	59.30 ± 7.81	0.090
Sex (female) (%)	69.6	78.1	50.0	0.105
BMI (kg/m^2^)	23.89 ± 3.67	23.38 ± 3.46	24.33 ± 4.68	0.700
HbA1c (%)	5.31 ± 0.35	5.86 ± 0.38^a^	8.73 ± 1.74^a,b^	<0.001
FPG (mmol/L)	5.24 ± 0.35	5.60 ± 0.61	8.66 ± 3.09^a,b^	<0.001
BUN (mmol/L)	5.72 ± 1.11	5.73 ± 1.53	6.18 ± 1.56	0.483
Cr (*μ*mol/L)	68.26 ± 19.13	62.06 ± 14.77	63.80 ± 16.99	0.399
UA (*μ*mol/L)	342.48 ± 113.33	338.59 ± 75.40	318.60 ± 76.59	0.648
Total cholesterol (mmol/L)	5.37 ± 0.89	5.75 ± 1.13	5.35 ± 1.27	0.320
Triglycerides (mmol/L)	1.60 ± 0.88	1.91 ± 0.98	1.65 ± 1.08	0.279

Abbreviations: BMI: body mass index; BUN: blood urea nitrogen; Cr: creatinine; FPG: fasting plasma glucose; UA: uric acid.

^a^Significant difference compared with controls.

^b^Significant difference compared with prediabetic patients.

**Table 2:** Corneal parameters in controls, prediabetic patients, and Type 2 diabetic patients (mean ± SD).

	**Controls**	**Prediabetes**	**Type 2 diabetes**	**p** ** value**
Eyes (no.)	40	60	40	—
CNFL (mm/mm^2^)	21.87 ± 3.04	18.64 ± 1.85^a^	17.27 ± 1.97^a,b^	<0.0001
CNFD (no./mm^2^)	149.58 ± 13.03	144.62 ± 7.92^a^	137.66 ± 11.25^a,b^	<0.0001
DC density (no./mm^2^)	10.93 ± 2.60	12.66 ± 2.02^a^	13.09 ± 1.77^a^	<0.0001
Corneal sensitivity (mm)	57.15 ± 2.29	54.98 ± 5.13	51.84 ± 7.02^a,b^	0.0004

Abbreviations: CNFD: corneal nerve fiber density; CNFL: corneal nerve fiber length; DC: dendritic cell.

^a^Significant difference compared with controls.

^b^Significant difference compared with prediabetic patients.

**Table 3:** Multivariable regression analysis for CNFL and risk factors.

	**Prediabetes**		**Type 2 diabetes**	
	**β**	**p** ** value**	**β**	**p** ** value**
Age (years)	−0.356	0.001	−0.474	0.010
BMI (kg/m^2^)	0.531	<0.001	−0.280	0.093
HbA1c (%)	0.033	0.755	−0.922	<0.001
FPG (mmol/L)	0.373	0.001	0.281	0.082
BUN (mmol/L)	−0.200	0.108	−0.585	<0.001
Cr (*μ*mol/L)	0.223	0.064	−0.474	0.031
UA (*μ*mol/L)	−0.303	0.027	−0.141	0.376
Total cholesterol (mmol/L)	0.203	0.067	−0.272	0.060
Triglycerides (mmol/L)	0.05	0.658	0.422	0.024

Abbreviations: BMI: body mass index; BUN: blood urea nitrogen; Cr: creatinine; FPG: fasting plasma glucose; UA: uric acid.

## Data Availability

The data supporting this study's findings are available upon reasonable request.
